# An end-to-end implicit neural representation architecture for medical volume data

**DOI:** 10.1371/journal.pone.0314944

**Published:** 2025-01-03

**Authors:** Armin Sheibanifard, Hongchuan Yu, Zongcai Ruan, Jian J. Zhang

**Affiliations:** 1 NCCA, Bournemouth University, Poole, United Kingdom; 2 Key Laboratory of Child Development and Learning Science, South-East University, Nanjing, China; Shandong Normal University, CHINA

## Abstract

Medical volume data are rapidly increasing, growing from gigabytes to petabytes, which presents significant challenges in organisation, storage, transmission, manipulation, and rendering. To address the challenges, we propose an end-to-end architecture for data compression, leveraging advanced deep learning technologies. This architecture consists of three key modules: downsampling, implicit neural representation (INR), and super-resolution (SR). We employ a trade-off point method to optimise each module’s performance and achieve the best balance between high compression rates and reconstruction quality. Experimental results on multi-parametric MRI data demonstrate that our method achieves a high compression rate of up to 97.5% while maintaining superior reconstruction accuracy, with a Peak Signal-to-Noise Ratio (PSNR) of 40.05 dB and Structural Similarity Index (SSIM) of 0.96. This approach significantly reduces GPU memory requirements and processing time, making it a practical solution for handling large medical datasets.

## 1 Introduction

Medical visualisation commonly involves volumetric medical data such as CT, MRI, PET scans, and confocal spectral microscopy images. This technique is essential in clinical practices across various biomedical disciplines, like radiology, nuclear medicine, surgery planning, and nearly all neuroscience sub-fields. However, the generated volume data often reaches enormous sizes. The generated data often becomes very large, sometimes reaching terabyte-scale. For instance, biological volumetric datasets that capture microscale details of cells or tissues are commonly produced [1–5]. The emerging challenges lie in organising, storing, transmitting, manipulating, and rendering such terabyte-scale volume data.

Recent advances in deep neural networks have led to their rapid application in medical imaging [6–8]. In particular, implicit neural representations have become an approach for compressing volumetric medical images by storing the parameters of trained neural networks instead of explicit voxel data such as SIREN [9]. However, the compression rate is often limited and volumetric data still require considerable memory, especially GPU memory. This results in high memory demands and longer training times for deep learning applications. In addition, there is currently a scarcity of research addressing these specific challenges.

To address these challenges, this paper presents an End-to-End architecture that improves compression rates and reduces GPU memory usage, based on our previous work [10]. The proposed architecture consists of three key modules: a downsampling module, an Implicit Neural Representation (INR) module, and a 3D Super-Resolution (SR) module (e.g., [11]). The downsampling module reduces data size, enabling the INR module to represent the volume using a compact deep neural network. The SR module then reconstructs the original high-resolution volume from the INR module output. This architecture reduces memory needs and allows for more efficient neural network training. The main challenge lies in achieving a high compression rate and minimal reconstruction loss. To address this, we propose a trade-off point method that optimises the configuration of each module to achieve peak performance. This approach can be generalised to a wide range of deep network designs. Our key contributions include:

- We propose an End-to-End architecture with three computational modules, designed to optimise volumetric data compression by achieving a high compression rate while maintaining superior reconstruction quality and minimising GPU memory consumption.
- We introduce a trade-off point method to determine the optimal configuration for the proposed End-to-End architecture, balancing key performance metrics such as compression rate and reconstruction quality.

The rest of the paper is structured as follows. Section 2 briefly reviews related work. Section 3 presents the proposed architecture and the trade-off point method. Section 4 presents experimental results and analysis. Finally, Section 5 concludes our work.

## 2 Background and relevant literature

In our previous work [10], we developed an architecture that leveraged existing pre-trained deep networks to decrease the volume data size. The basic idea is to transform volume data into an implicit neural network representation, such as SIREN [9], to compress the data while maintaining reconstruction accuracy. However, pre-trained deep networks often struggle to generalise well, especially with medical volume data. Many pre-trained Super-Resolution deep networks require fine-tuning for different medical datasets. A “one-size-fits-all” approach does not work, since each dataset has its own characteristics. The existing deep networks do not generalise well to diverse volume data. Therefore, this paper aims to train an end-to-end deep network, rather than simply piecing together multiple pre-trained networks.

### 2.1 Implicit neural representation

Representing 3D geometry for rendering and reconstruction involves trade-offs across fidelity, efficiency, and compression capabilities. The DeepSDF model [12] uses a continuous Signed Distance Function (SDF) to represent shapes. Another approach [13] employs an encoder-decoder neural architecture for lossless compression. However, this method has a high inference time due to explicit optimisation requirements.

MedZip [14] proposes a lossless compression technique employing Long Short-Term Memory (LSTM) for volumetric MRI and CT. NeRF [15] presents a notable method for synthesising new views of a volumetric scene through implicit neural representation as a continuous function. However, it is outperformed by SIRENs [9] due to its time consumption. [16] presents a 3D representation technique to reduce memory usage by predicting an occupancy function for a continuous volume. COIN [17] applies a multi-layer perceptron (MLP) to implicit neural network compression by encoding geometric inputs. However, it demonstrates inferior performance compared to state-of-the-art compression methods. INR-GAN [18] applies a GAN model to multi-scale Implicit Neural Representations (INRs) but struggles with artefacts when dealing with high-frequency features. NeRP [19] introduces a novel approach to generate a computational image from sampled sensor data. However, dealing with sparsely sampled images encounters additional hurdles due to limited data points. Unlike previous deep learning methods for image reconstruction, NeRP leverages both the internal structure of an image prior and the physics governing sparsely sampled measurements to represent the entire subject.

### 2.2 Super-resolution techniques

Numerous techniques leveraging convolutional neural networks (CNNs) have demonstrated exceptional performance in image super-resolution (SR). The pioneering work of SRCNN [20] introduced CNNs to SR by learning a non-linear mapping from low-resolution to high-resolution images with only three convolution layers. CNN-based methods illustrated their impressive performance in SR. Still, they became impractical when taking into account constraints on time and memory resources [21–30]. SRNO [11] designed for continuous super-resolution tasks. It treats each image as a function and learns a mapping between finite-dimensional function spaces, enabling it to train and generalise across various discretisation levels. Experiments demonstrate that SRNO surpasses other arbitrary-scale super-resolution methods in terms of both performance and computational time, particularly excelling in capturing global image structures, which is important in medical imaging.

Table 1 highlights the gaps between the proposed method and four state-of-the-art models—SIREN [9], MedZip [14], NeRF [15], and COIN [17]—across several key metrics: high compression rate, low GPU memory consumption, high reconstruction quality (PSNR > 40), good visual similarity (SSIM > 0.9), scalability to large datasets, fast training time, adaptability to medical imaging, and handling high-frequency features. The proposed method addresses several limitations of existing models, particularly in achieving high compression rates and excellent reconstruction quality, while maintaining efficiency in GPU memory usage and adaptability to medical imaging tasks.

**Table 1:** Identifying gaps in state-of-the-art models compared to the proposed method.

Feature/Metric	Proposed Method	SIREN [9]	MedZip [14]	NeRF [15]	COIN [17]
High Compression Rate	✓	✓			✓
Low GPU Memory Consumption	✓	✓			
High Reconstruction Quality (PSNR > 40)	✓		✓	✓	
Good Visual Similarity (SSIM > 0.9)	✓	✓		✓	
Scalable to Large Datasets	✓			✓	
Fast Training Time	✓	✓			
Adaptability to Medical Imaging	✓		✓		
Handles High-Frequency Features Well	✓	✓		✓	✓

## 3 Methodology

In this section, we first present the end-to-end architecture and then introduce the trade-off point approach to evaluate the proposed architecture in terms of compression efficiency and reconstruction accuracy.

### 3.1 Proposed end-to-end architecture

**Fig 1:**
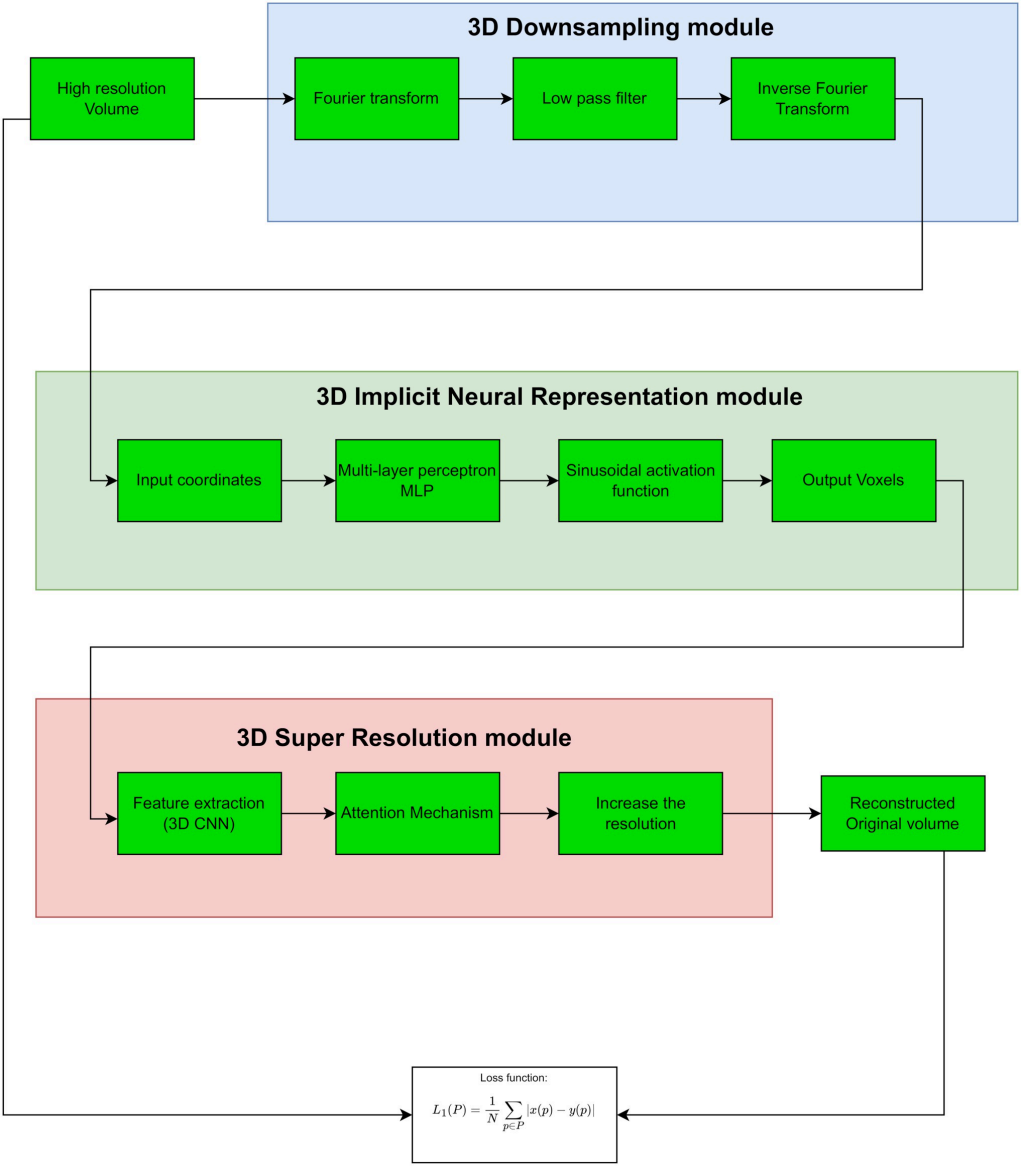
Workflow of the proposed end-to-end architecture, including downsampling, implicit neural representation (INR), and super-resolution (SR) modules.

Our end-to-end architecture, shown in Fig 1, is composed of three core modules: Downsampling, Implicit Neural Representation (INR), and Super-Resolution (SR). The Downsampling module does not require training. We need to train the INR and SR modules in an end-to-end way. We employ a *L*_1_ loss function to evaluate reconstruction quality here. In the following sections, we will explain each module individually.

#### 3.1.1 3D downsampling module

Given a high-resolution volume of *x*, this module aims to acquire its low-resolution counterpart y. The relationship between x and y can be modelled as follows,

$$\begin{array}{c} y=F_{LR}^{-1}DF_{HR}x+n \end{array}$$ (1)

where, $F_{HR}$ is the FFT operator for the high-resolution regime, $F_{LR}^{-1}$ is the inverse FFT operator for the low-resolution regime, D is the low-pass operator on the frequency domain, and *n* is the noise. Fourier Transform technique is widely employed in medical imaging [31]. We hope to point out that the operator D in the frequency domain is both controllable and easy to implement. In our case, it effectively generates low-resolution volumes at downsampling scales of $\times \frac{1}{2}$, $\times \frac{1}{4}$, and $\times \frac{1}{8}$. Additionally, it can be noted that this module does not need training.

#### 3.1.2 3D implicit neural representation (INR)

The INR module harnesses the capabilities of implicit neural networks to efficiently encode volumetric data. Specifically, using INR for low-resolution volumes helps prevent memory overflow. Unlike conventional explicit representations, INRs depict the volume as a continuous function that maps spatial coordinates to voxel intensity values. This enables a concise representation that can be readily adjusted to different levels of detail. Drawing inspiration from recent breakthroughs in implicit neural representations, we employed a multi-layer perceptron (MLP) architecture with periodic activation functions (i.e., SIREN [9]) to effectively capture the intricate structures within the volumetric data.

#### 3.1.3 3D super resolution (SR) module

The SR module employs the super-resolution model, SRNO [11]. SRNO model utilises deep learning to learn intricate transformations from low-resolution to high-resolution data. Beyond enhancing resolution, SRNO models frequently possess intrinsic denoising abilities, resulting in cleaner and clearer images. Compared to other super-resolution techniques, SRNO models can produce images with fewer artefacts, such as ringing and blurring [11]. Moreover, the number of channels in the attention structure can significantly influence the SRNO model’s performance. Thus, we regard it as a hyper-parameter of the SRNO models and evaluate the SRNO by it.

### 3.2 Trade-off point approach

To achieve an overall optimal performance for our proposed end-to-end architecture, we propose a metric system to measure overall performance and further determine the optimal setting for each module accordingly. This design method is called the Trade-off Point Method. Our metric system includes four measurements: Peak Signal-to-Noise Ratio (PSNR), Structural Similarity Index (SSIM), Bitrate, and Compression Rate (CR) as below. PSNR provides a measure of pixel-level accuracy by calculating the ratio of signal power to noise power, yet it often does not correspond to human visual perception. In contrast, SSIM assesses perceptual quality by comparing luminance, contrast, and structure, but may overlook precise pixel-wise errors. Recognising the limitations of using PSNR or SSIM alone for performance measurement, we combine both metrics to evaluate image quality thoroughly.

#### 3.2.1 Metric definition

- **Peak Signal to Noise Ratio(PSNR)** is a metric used to measure the quality of a reconstructed or compressed signal compared to the original signal. It is expressed in decibels (dB) and is calculated using the following formula:
  $$\begin{array}{c} \text{PSNR}=10\cdot {\text{log}}_{10}(\frac{{\text{MAX}}^{2}}{\text{MSE}}) \end{array}$$ (2)
  where: MAX is the maximum possible pixel value of the image (e.g., 255 for an 8-bit image), and MSE is the Mean-Squared Error between the original and reconstructed images.
  A high PSNR value indicates a high-quality reconstruction, as it signifies that the reconstructed signal is closer to the original signal in terms of fidelity.
- **Structural Similarity Index Measurement(SSIM):** The Structural Similarity Index Measurement(SSIM) is a metric to assess the similarity between a reference image (original) and a distorted or processed image. SSIM quantifies similarity by considering three key components: luminance, contrast, and structure. SSIM is defined as,
  $$\begin{array}{c} \text{SSIM}\left(x,y\right)=\frac{\left(2\mu_{x}\mu_{y}+C_{1}\right)\left(2\sigma_{xy}+C_{2}\right)}{\left(\mu_{x}^{2}+\mu_{y}^{2}+C_{1}\right)\left(\sigma_{x}^{2}+\sigma_{y}^{2}+C_{2}\right)} \end{array}$$ (3)
  where: *μ*_*x*_ and *μ*_*y*_ are the means of the original and distorted images, respectively, $\sigma_{x}^{2}$ and $\sigma_{y}^{2}$ are the variances of the original and distorted images, respectively, *σ*_*xy*_ is the covariance of the original and distorted images, *C*_1_ and *C*_2_ are small constants added for numerical stability. The SSIM value ranges from -1 to 1, with 1 indicating perfect similarity. High SSIM values indicate high similarity between the images, while low values suggest more significant differences or distortions.
- **Bitrate:** Bitrate is a metric used in digital imaging to quantify the amount of data assigned to each pixel in a raster image. Bpp indicates the level of detail or precision in representing colour or intensity information for each pixel. High Bpp values typically result in high image quality but large file size, while low Bpp values lead to low quality but small files. It is computed as,
  $$\begin{array}{c} \text{Bitrate}=\frac{\text{Total}\;\text{bits}}{\text{Total}\;\text{pixels}} \end{array}$$ (4)
  In greyscale images, each pixel is represented by a single channel (e.g., luminance). Bpp is degraded as,
  $$\begin{array}{c} \text{Bitrate}=\frac{\text{Bit}\;\text{depth}}{1} \end{array}$$ (5)
  When compression techniques are applied, the Bitrate measures the density of the pixel value of the image to assess the trade-off between image quality and file size. High Bitrate values generally result in high-quality but large image files, while low Bitrate values lead to more aggressive compression and small files but with potential quality loss.
- **Downsampling Scale (DS):** Let *D*_*x*_, *D*_*y*_, and *D*_*z*_ be the original dimensions of the 3D image stacks in a (*x*, *y*, *z*) coordinate system, respectively; and the new dimensions be (*d*_*x*_, *d*_*y*_, *d*_*z*_) after downsampling. The *DS* (*s*_*x*_, *s*_*y*_, *s*_*z*_) is defined as,
  $$\begin{array}{c} d_{x}=\frac{D_{x}}{s_{x}},d_{y}=\frac{D_{y}}{s_{y}},d_{z}=\frac{D_{z}}{s_{z}} \end{array}$$
  We may simply set (*s*_*x*_, *s*_*y*_, *s*_*z*_) identically.
- **Number of the neurons in SIREN (SN):** With SIREN’s layer count set at 3, each layer contains an identical number of neurons. We adjust the neuron count per layer from 30 to 230, using this to represent SIREN’s size.
- **Number of Channels (NC):** We incorporate the 3D version of SRNO into the SR module. The cornerstone of a super-resolution network lies in its feature extractor. Existing super-resolution models possess their own topologies for their feature extractors. The number of Channels indicates the feature extractor’s size, thereby reflecting the complexity of the super-resolution network. This complexity is particularly influenced by the downsampling scale within our proposed architecture, leading to a significant increase in channel numbers due to the abundance of volume data. To minimise the size of the SR module in our proposed architecture, we initially assess the performance of the SR module with different sizes of attention mechanisms and fully connected layer submodules, after which we fix the topologies and sizes of these two submodules. However, the channel number of the feature extractor remains adaptable to accommodate varying reconstruction accuracy requirements.
- **Compression Rate (CR):** The CR refers to the ratio of the compressed data’s size over the uncompressed data’s size. A high compression rate indicates an efficient compression process, as it signifies a remarkable reduction in data size. It is defined as,
  $$\begin{array}{c} CR=(1-\frac{\text{Size}\;\text{of}\;\text{the}\;\text{network}}{\text{Size}\;\text{of}\;\text{Uncompressed}\;\text{Data}})\times 100\% \end{array}$$ (6)
  In this paper, we define the size of a deep network by its weight count and the size of a volume by its voxel number.

#### 3.2.2 Trade-off settings

**Fig 2:**
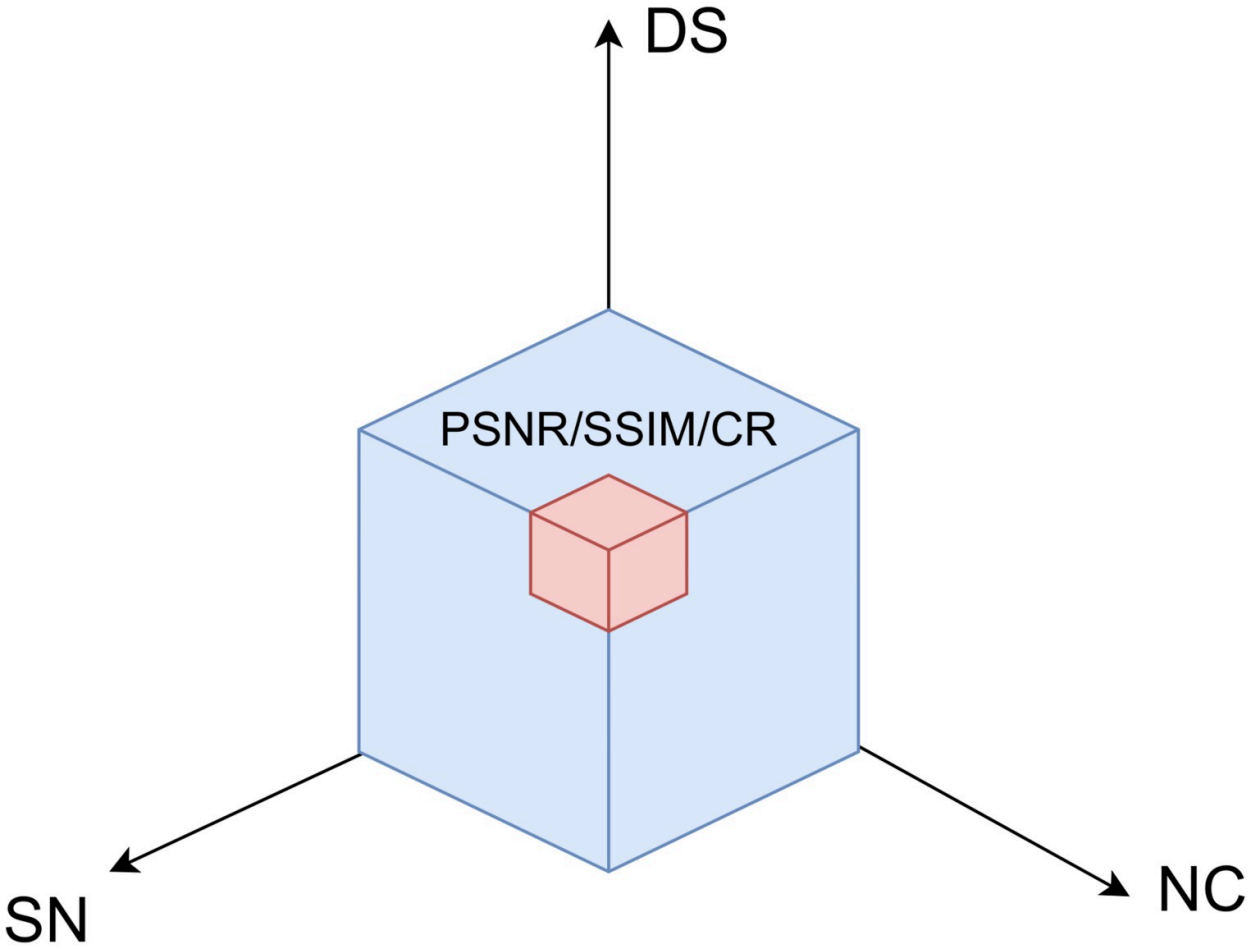
Illustration of the data structure in the context of the metrics, PSNR, SSIM and CR, according to the DS, NC and SN dimensions.

To find the trade-off settings for the individual modules, we first apply the metrics of PSNR, SSIM, and CR defined in the above section separately to a specific volume of data concerning three dimensions: DS, NC, and SN. The different combinations of DS, NC, and SN result in different measurements, which are stored in a 3D array, as shown in Fig 2. We need to balance the performance of (PSNR, SSIM, and CR) associated with the combination of three dimensions (DS, NC, SN) to determine the trade-off point for our end-to-end architecture. This may be described as,

$$\begin{array}{c} \left\{ \begin{array}{c} \min_{(x,y,z)\in 3DA}(\frac{1}{\text{PSNR}}+1-\left|\text{SSIM}\right|+(1-\text{CR}))\\ \text{subject}\;\text{to}\{\begin{array}{cc} c_{1}: & x-{\text{DS}}_{\text{max}}=0\\ c_{2}: & y-{\text{NC}}_{\text{min}}=0\\ c_{3}: & z-{\text{SN}}_{\text{min}}=0 \end{array} \end{array}\right. \end{array}$$ (7)

where, 3DA denotes the 3D array with 3 dimensions, DS, NC, SN, and *DS*_*max*_ denotes the given maximum value for DS, and others have a similar definition. Applying the Augmented Lagrangian method here yields,

$$\begin{array}{c} \text{TradeOff}=\underset{(x,y,z)\in 3DA}{\text{argmin}}(\frac{1}{\text{PSNR}}+1-\left|\text{SSIM}\right|+(1-\text{CR})-\sum_{i=1}^{3}\alpha_{i}c_{i}+\frac{1}{2}\beta \sum_{i=1}^{3}c_{i}^{2}) \end{array}$$ (8)

where *α* are Lagrange factors and *β* is the penalty parameter. The resulting (x,y,z) is called the trade-off point. To visualise it, we compute the marginal distributions concerning three dimensions separately on 3DA as below,

$$\begin{array}{c} \left\{ \begin{array}{c} PSNR\left(x∼3DA\left(DS\right)\right)=\sum_{\left(y,z)∼3DA(NC,SN\right)}PSNR\left(x,y,z\right)\\ SSIM\left(x∼3DA\left(DS\right)\right)=\sum_{\left(y,z)∼3DA(NC,SN\right)}SSIM\left(x,y,z\right)\\ CR\left(x∼3DA\left(DS\right)\right)=\sum_{\left(y,z)∼3DA(NC,SN\right)}CR\left(x,y,z\right) \end{array}\right. \end{array}$$ (9)

There are a total of three sets of marginal distributions. Each set illustrates the PSNR bounds, SSIM bounds, and CR bounds concerning the scale at each dimension specified by the trade-off point, one after another. Theoretical equivalence is expected among these three sets of PSNR, SSIM and CR bounds at the trade-off point. The trade-off point indicates the tolerance of the proposed architecture in three dimensions at an expected PSNR, SSIM and CR bounds level. The area delimited by the trade-off point intuitively and quantitatively illustrates the proposed architecture’s performance.

## 4 Materials and experimental results

Our experiments can be categorised into two parts. The first part aims to justify the selection of each module in our proposed end-to-end architecture. The second part involves applying the trade-off point method to determine an optimal architecture that balances various considerations.

### 4.1 Data and implementation setup

The dataset comprises 750 multi-parametric magnetic resonance images (mp-MRI) collected from patients diagnosed with either glioblastoma or lower-grade glioma [32]. We select T2 Fluid-Attenuated Inversion Recovery (FLAIR) 3D scan from a random patient with the size of 155 x 240 x 240. The implementation of our architecture starts with a high-resolution 3D volumetric input, such as a medical scan, denoted as *x*. Initially, the input volume undergoes normalisation, scaling the voxel values to a range between 0 and 1. To streamline computations, the volume is segmented into smaller patches, each measuring 64 × 64 × 64. Patches with 70% or more non-zero voxels containing more information are classified as High-Resolution (HR) patches. From these, one HR patch is selected as the high-resolution input for further processing.

Once the data are prepared, the 3D Downsampling module applies a Fourier Transform to convert the high-resolution volume from the spatial domain to the frequency domain. A low-pass filter is then used to eliminate high-frequency components, thereby reducing resolution. This removal process is crucial in medical imaging, as it decreases the data size while preserving essential information, ultimately easing the model processing load. The Inverse Fourier Transform reverts the data to the spatial domain, yielding a low-resolution version of the original volume.

Next, the downsampled volume is processed through the 3D Implicit Neural Representation (INR) module. Here, a Multi-Layer Perceptron (MLP) utilising Sinusoidal Activation Functions (SIREN) maps input coordinates to output voxel intensities, enabling the neural network to represent complex structures as continuous functions. These functions are then converted into voxel intensities.

Following this, the 3D Super-Resolution (SR) module employs a 3D Convolutional Neural Network (CNN) for feature extraction, incorporating an Attention Mechanism to prioritise significant features. This SR module improves the resolution of the volume, restoring it to a level close to the original.

The reconstructed volume, denoted as *y*, is compared to the original *x* using an *L*_1_ loss function to assess and optimise reconstruction quality. The entire system is trained using the Adam optimiser with a learning rate of 0.0015 for 5,000 epochs on an NVIDIA A4000 16GB GPU with CUDA support in the PyTorch framework. All source codes and results are available at https://github.com/asheibanifard/EndtoEndCompression.

### 4.2 Trade-off architecture

#### 4.2.1 3D downsampling module

The Downsampling module does not require training. This implies that the downsampling scale is per set without consideration of the final result quality. We select three downsampling scales of 1/2, 1/4, and 1/8 in our experiments. It is necessary to test the performance of the proposed architecture at three downsampling scales, particularly the INR module. Table 2 presents a comprehensive comparison of reconstruction results for different downsampling scales, illustrating the effectiveness of our proposed architecture in maintaining a high reconstruction quality across various compression levels. It can be noted that decreasing the downsampling scales does not significantly degenerate the quality of the reconstruction. Additionally, non-standard sampling scales like 1/3, 1/5, or 1/7 would introduce unnecessary complexity and inconsistencies without offering meaningful improvements, making them less suitable for the architecture’s goals. Thus, these three downsampling scales are acceptable.

**Table 2:** Performance of the INR module and the whole end-to-end architecture. (The upper row shows the performance of a single SIREN and the lower row shows that of the whole end-to-end architecture).

INR module					
Scale	Avg Bitrate ↓	Avg CR (%)↑	Avg PSNR ↑	Avg SSIM ↑	Avg #Para ↓
1/2	5.21	83.71	36.96	0.95	42711
1/4	5.21	83.71	51.48	1.00	42711
1/8	5.21	83.71	67.34	1.00	42711
Whole end-to-end Architecture					
Scale	Avg Bitrate ↓	Avg CR (%)↑	Avg PSNR ↑	Avg SSIM ↑	Avg #Para ↓
1/2	5.743	82.052	38.001	0.956	47048.0
1/4	6.655	79.200	38.381	0.953	54524.0
1/8	10.062	68.553	39.462	0.961	82436.0

#### 4.2.2 3D INR module

We opt for the SIREN model [9] as our INR module, focusing on two primary aspects of the SIREN structure: the number of layers and the number of neurons per layer. The goal is to use a compact SIREN model to enhance the compression rate (CR). We experiment with various configurations of the SIREN model, altering the layer count and neuron count per layer, as detailed in Table 3. We find that a SIREN network with 3 layers and between 30 and 230 neurons per layer offers satisfactory performance, especially for small volume data inputs, while substantially cutting down on GPU memory usage. Furthermore, we compare the performance of a single SIREN model against our proposed architecture, as shown in Table 2. The notable benefit is a dramatic reduction in GPU memory consumption while maintaining comparable reconstruction quality. Additionally, using more than 230 neurons per layer increases the model’s capacity to represent detailed structures but leads to diminishing returns in terms of reconstruction quality. Beyond 230 neurons, the gains in PSNR and SSIM are marginal, while the computational cost and GPU memory usage increase significantly. This increased complexity does not translate into substantial improvements in performance, making the additional computational overhead unjustified. Thus, we prefer the SIREN model with 3 layers in the INR module.

**Table 3:** Average values for different INR layers and neurons.

Layers	Neurons	Bitrate(bpp) ↓	CR (%)↑	PSNR ↑	SSIM ↑	#Para ↓
3	30	0.245	99.233	31.081	0.767	2011
3	50	0.653	97.959	32.205	0.804	5351
3	70	1.256	96.074	34.550	0.903	10291
3	90	2.055	93.579	35.637	0.923	16831
3	110	3.048	90.474	36.610	0.942	24971
3	130	4.237	86.759	37.862	0.960	34711
3	150	5.621	82.433	38.389	0.964	46051
3	170	7.201	77.497	38.626	0.965	58991
3	190	8.976	71.950	39.954	0.975	73531
3	210	10.946	65.793	39.553	0.974	89671
3	230	13.112	59.026	40.934	0.981	107411
4	30	0.359	98.878	29.008	0.627	2941
4	50	0.964	96.986	32.472	0.825	7901
4	70	1.863	94.178	34.814	0.902	15261
4	90	3.054	90.455	36.450	0.937	25021
4	110	4.539	85.817	37.251	0.951	37181
4	130	6.316	80.262	39.872	0.974	51741
4	150	8.386	73.793	41.887	0.984	68701
4	170	10.750	66.407	42.395	0.986	88061
4	190	13.406	58.107	42.738	0.988	109821
4	210	16.355	48.890	43.586	0.989	133981
4	230	19.597	38.758	44.335	0.991	160541
5	30	0.473	98.523	30.846	0.781	3871
5	50	1.276	96.013	32.469	0.799	10451
5	70	2.470	92.282	38.391	0.964	20231
5	90	4.054	87.331	38.456	0.963	33211
5	110	6.029	81.159	40.037	0.976	49391
5	130	8.395	73.766	41.990	0.985	68771
5	150	11.151	65.152	42.953	0.988	91351
5	170	14.298	55.318	42.284	0.986	117131
5	190	17.836	44.263	43.366	0.989	146111
5	210	21.764	31.987	44.676	0.991	178291
5	230	26.083	18.491	44.616	0.991	213671
6	30	0.586	98.169	30.077	0.774	4801
6	50	1.587	95.041	36.246	0.935	13001
6	70	3.076	90.387	39.529	0.974	25201
6	90	5.054	84.207	40.204	0.977	41401
6	110	7.520	76.501	41.779	0.984	61601
6	130	10.474	67.270	41.733	0.984	85801
6	150	13.916	56.512	43.195	0.988	114001
6	170	17.847	44.229	42.773	0.988	146201
6	190	22.266	30.420	44.301	0.991	182401
6	210	27.173	15.084	42.274	0.984	222601
6	230	32.568	-1.777	43.441	0.988	266801

#### 4.2.3 3D super-resolution module

We utilise the SRNO [11] for the SR module due to its compact size, as evidenced by the average number of parameters of deep networks in Table 2. We also compare our end-to-end architecture with cutting-edge methods [32–37]. Table 8 reveals that (1) the SR module performs effectively, as our architecture, using a 3-layer SIREN, matches the reconstruction quality of a standalone 5-layer SIREN; and (2) our architecture surpasses other state-of-the-art image compression methods in terms of PSNR and SSIM.

#### 4.2.4 Find a trade-off architecture by trade-off point approach

To find the trade-off point for our proposed architecture, firstly, our proposed architecture is tested in terms of all combinations of NC, DS and SN, which is presented separately in Table 4 with 4 channels of feature extraction in the SRNO model, Table 5 with 8 channels of feature extraction in the SRNO model, and Table 6 with 16 channels of feature extraction in the SRNO model. The trade-off point of the proposed architecture is then calculated using Eq 8, that is, the trade-off point (NC = 4, DS = 1/2, SN = 30). At the trade-off point, the PSNR upper bound is around 38, the SSIM upper bound is around 0.94, and the CR upper bound is around 76.6%, as shown in Table 7. This is a good setting for the proposed architecture, as it reaches a high compression rate and good quality for reconstruction.

**Table 4:** The results of our proposed architecture with 4 channels of shallow feature extractor in SR module.

Scale	# Neurons	Bitrate(bpp) ↓	CR(%)↑	PSNR(db) ↑	SSIM ↑	#Para ↓	GPU memory(GB) ↓
1/2	30	0.775	97.578	33.885	0.885	6348	1.366
1/2	50	1.183	96.304	35.211	0.915	9688	1.395
1/2	70	1.786	94.420	36.853	0.947	14628	1.426
1/2	90	2.584	91.925	37.682	0.961	21168	1.456
1/2	110	3.578	88.820	38.547	0.969	29308	1.484
1/2	130	4.767	85.104	38.968	0.972	39048	1.512
1/2	150	6.151	80.779	38.867	0.974	50388	1.540
1/2	170	7.730	75.842	39.419	0.973	63328	1.570
1/2	190	9.505	70.296	39.603	0.976	77868	1.599
1/2	210	11.476	64.139	39.319	0.970	94008	1.629
1/2	230	13.641	57.372	39.665	0.976	111748	1.661
1/4	30	1.520	95.250	33.221	0.858	12452	1.319
1/4	50	1.928	93.976	33.985	0.892	15792	1.322
1/4	70	2.531	92.091	34.503	0.916	20732	1.331
1/4	90	3.329	89.597	34.789	0.921	27272	1.335
1/4	110	4.323	86.491	34.753	0.915	35412	1.333
1/4	130	5.512	82.776	34.825	0.921	45152	1.349
1/4	150	6.896	78.450	35.080	0.923	56492	1.348
1/4	170	8.476	73.514	35.001	0.924	69432	1.347
1/4	190	10.250	67.967	34.977	0.919	83972	1.347
1/4	210	12.221	61.810	35.300	0.927	100112	1.354
1/4	230	14.386	55.043	35.393	0.922	117852	1.355
1/8	30	7.481	76.622	40.991	0.977	61284	1.313
1/8	50	7.889	75.348	36.212	0.909	64624	1.314
1/8	70	8.492	73.463	40.873	0.977	69564	1.312
1/8	90	9.290	70.969	38.934	0.965	76104	1.315
1/8	110	10.284	67.863	40.995	0.979	84244	1.319
1/8	130	11.473	64.148	40.799	0.978	93984	1.316
1/8	150	12.857	59.822	40.150	0.975	105324	1.318
1/8	170	14.437	54.886	39.587	0.974	118264	1.316
1/8	190	16.211	49.339	39.866	0.973	132804	1.318
1/8	210	18.182	43.182	38.954	0.966	148944	1.317
1/8	230	20.347	36.415	39.094	0.960	166684	1.319

**Table 5:** The results of our proposed network with 8 channels of shallow feature extractor in SR module.

Scale	# Neurons	Bitrate(bpp) ↓	CR(%)↑	PSNR(db) ↑	SSIM ↑	#Para ↓	GPU memory(GB) ↓
1/2	30	1.688	94.727	32.829	0.825	13824	1.330
1/2	50	2.095	93.452	36.009	0.922	17164	1.360
1/2	70	2.698	91.568	37.112	0.945	22104	1.387
1/2	90	3.497	89.073	38.502	0.964	28644	1.422
1/2	110	4.490	85.968	39.373	0.975	36784	1.451
1/2	130	5.679	82.253	39.127	0.974	46524	1.479
1/2	150	7.063	77.927	39.365	0.971	57864	1.506
1/2	170	8.643	72.990	41.106	0.982	70804	1.537
1/2	190	10.418	67.444	40.133	0.979	85344	1.562
1/2	210	12.388	61.287	38.495	0.976	101484	1.595
1/2	230	14.554	54.520	40.149	0.976	119224	1.625
1/4	30	3.171	90.091	35.348	0.886	25976	1.277
1/4	50	3.579	88.817	36.051	0.917	29316	1.281
1/4	70	4.182	86.932	37.443	0.941	34256	1.289
1/4	90	4.980	84.438	36.178	0.935	40796	1.291
1/4	110	5.974	81.332	38.016	0.948	48936	1.295
1/4	130	7.163	77.617	37.564	0.946	58676	1.307
1/4	150	8.547	73.291	38.021	0.948	70016	1.307
1/4	170	10.126	68.355	36.991	0.944	82956	1.307
1/4	190	11.901	62.808	38.424	0.950	97496	1.310
1/4	210	13.872	56.651	36.755	0.942	113636	1.311
1/4	230	16.037	49.884	36.696	0.942	131376	1.319
1/8	30	15.038	53.006	45.056	0.990	123192	1.273
1/8	50	15.446	51.732	44.628	0.989	126532	1.272
1/8	70	16.049	49.847	45.004	0.990	131472	1.276
1/8	90	16.847	47.353	44.226	0.987	138012	1.272
1/8	110	17.841	44.247	45.528	0.992	146152	1.277
1/8	130	19.030	40.532	43.596	0.985	155892	1.276
1/8	150	20.414	36.206	44.410	0.990	167232	1.276
1/8	170	21.994	31.270	43.539	0.986	180172	1.276
1/8	190	23.769	25.723	43.650	0.988	194712	1.277
1/8	210	25.739	19.566	42.061	0.981	210852	1.277
1/8	230	27.904	12.799	41.435	0.978	228592	1.275

**Table 6:** The results of our proposed network with 16 channels of shallow feature extractor in SR module.

Scale	# Neurons	Bitrate(bpp) ↓	CR(%)↑	PSNR(db) ↑	SSIM ↑	#Para ↓	GPU memory(GB) ↓
1/2	30	5.095	84.079	34.469	0.858	41736	1.366
1/2	50	5.502	82.805	37.843	0.947	45076	1.396
1/2	70	6.105	80.920	39.609	0.965	50016	1.424
1/2	90	6.904	78.426	39.634	0.967	56556	1.457
1/2	110	7.897	75.320	40.585	0.979	64696	1.486
1/2	130	9.086	71.605	38.699	0.975	74436	1.514
1/2	150	10.471	67.279	41.600	0.982	85776	1.546
1/2	170	12.050	62.343	39.853	0.969	98716	1.568
1/2	190	13.825	56.796	41.416	0.981	113256	1.601
1/2	210	15.795	50.639	41.327	0.977	129396	1.632
1/2	230	17.961	43.872	39.053	0.977	147136	1.660
1/4	30	8.055	74.829	35.132	0.891	65984	1.272
1/4	50	8.462	73.555	38.964	0.941	69324	1.279
1/4	70	9.065	71.671	40.981	0.965	74264	1.290
1/4	90	9.864	69.176	40.182	0.965	80804	1.289
1/4	110	10.857	66.071	40.705	0.969	88944	1.292
1/4	130	12.046	62.355	42.462	0.979	98684	1.298
1/4	150	13.431	58.029	42.580	0.981	110024	1.307
1/4	170	15.010	53.093	41.079	0.970	122964	1.308
1/4	190	16.785	47.546	42.322	0.979	137504	1.306
1/4	210	18.755	41.389	41.177	0.974	153644	1.311
1/4	230	20.921	34.622	41.987	0.979	171384	1.313
1/8	30	31.734	0.830	49.798	0.998	259968	1.270
1/8	50	32.142	-0.444	48.750	0.996	263308	1.271
1/8	70	32.745	-2.328	48.238	0.995	268248	1.271
1/8	90	33.543	-4.823	48.497	0.997	274788	1.271
1/8	110	34.537	-7.928	46.755	0.993	282928	1.280
1/8	130	35.726	-11.644	48.436	0.997	292668	1.275
1/8	150	37.110	-15.970	50.539	0.999	304008	1.277
1/8	170	38.690	-20.906	48.897	0.996	316948	1.273
1/8	190	40.465	-26.453	48.730	0.997	331488	1.275
1/8	210	42.435	-32.610	46.971	0.994	347628	1.277
1/8	230	44.601	-39.377	47.476	0.996	365368	1.277

**Table 7:** Our proposed architecture’s trade-off point.

Marginal values	NC = 4	DS = 1/2	SN = 30
1/PSNR	0.02598	0.02681	0.02694
1 − |*SSIM*|	0.04287	0.05491	0.09239
1 − *CR*	0.23398	0.25709	0.25887

**Fig 3:**
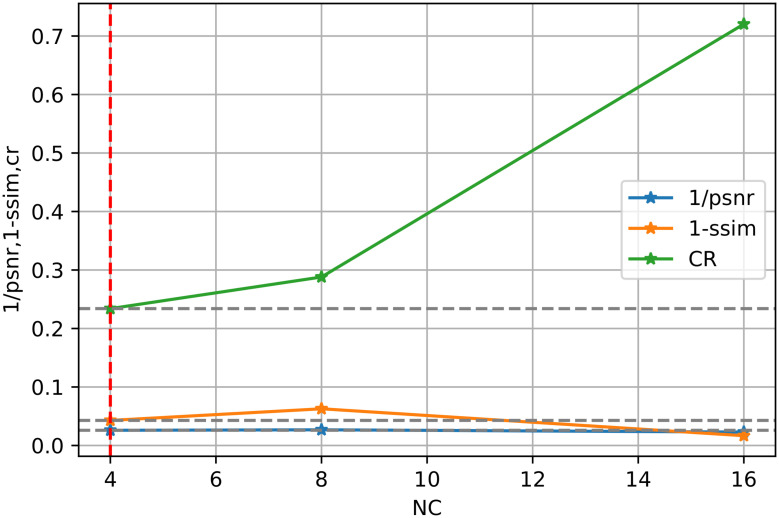
Illustrates the trade-off point for the number of channels (NC) in the SR module concerning the performance metrics, 1/PSNR, 1-SSIM, and 1-CR. The red dashed lines indicate the intersection where the optimal trade-off is achieved, balancing compression efficiency and reconstruction quality.

**Fig 4:**
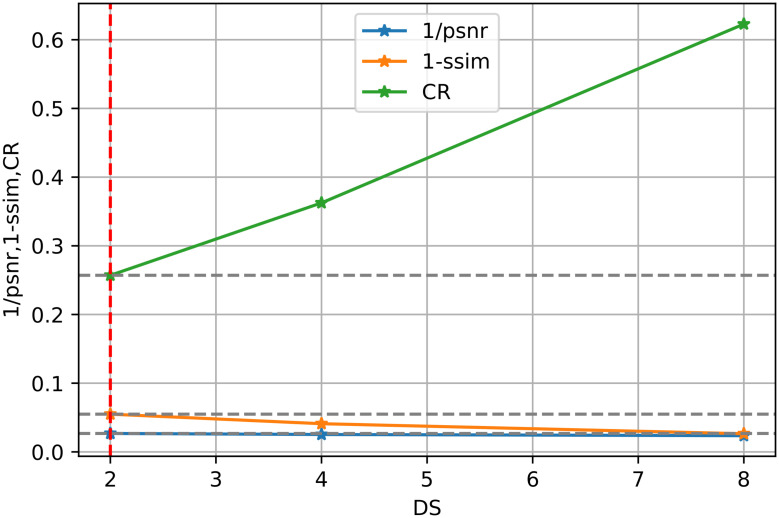
The trade-off point for the downsampling scale (DS) is based on the performance metrics, 1/PSNR, 1-SSIM, and 1-CR. The red dashed lines highlight where the downsampling scale achieves an optimal balance between compression rate and reconstruction accuracy.

**Fig 5:**
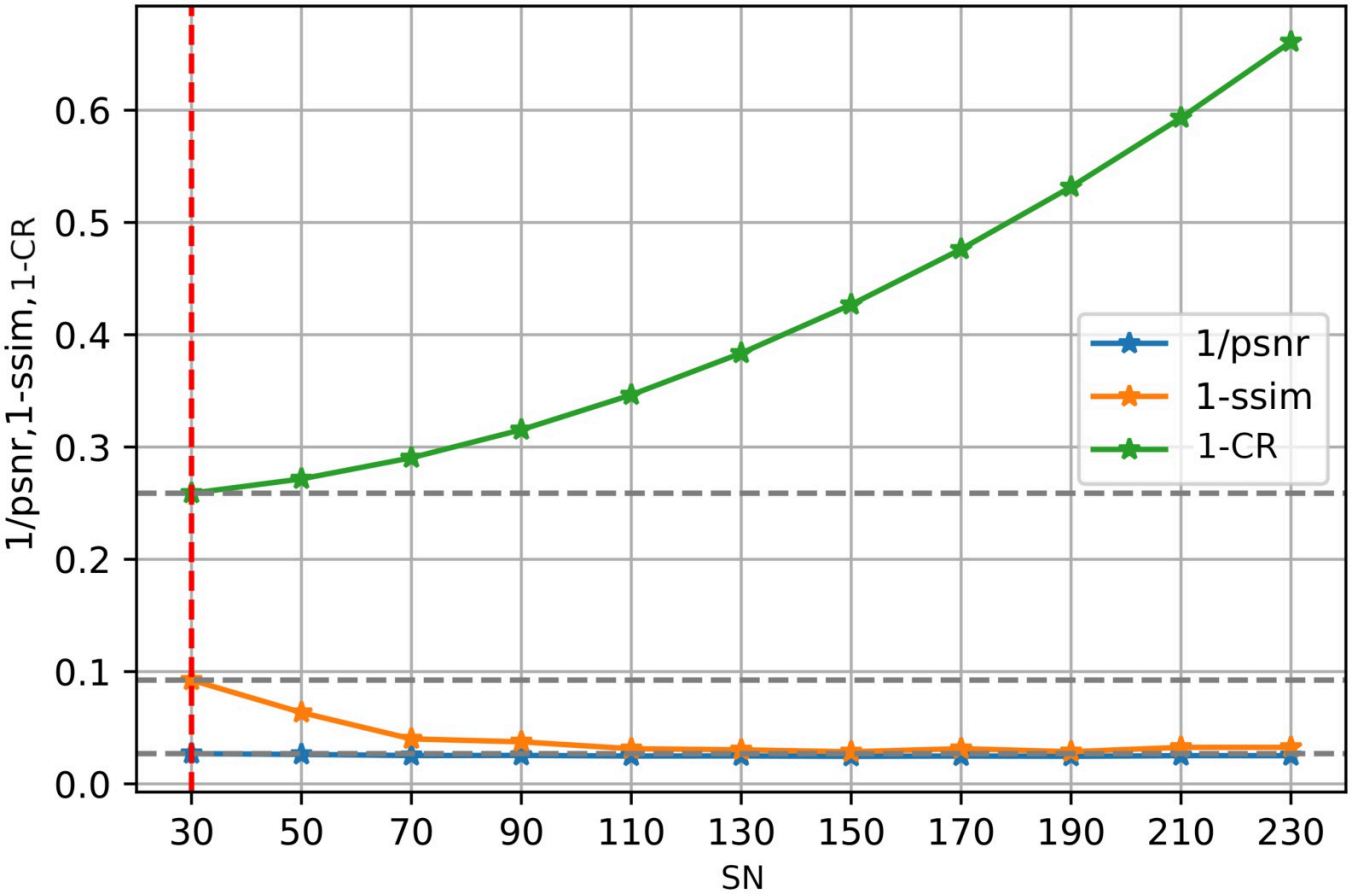
The trade-off point for the number of neurons (SN) in the SIREN model, plotted against the performance metrics, 1/PSNR, 1-SSIM, and 1-CR. The red dashed lines indicate the optimal configuration of neurons in the SIREN model for achieving high reconstruction quality with minimal compression loss.

**Fig 6:**
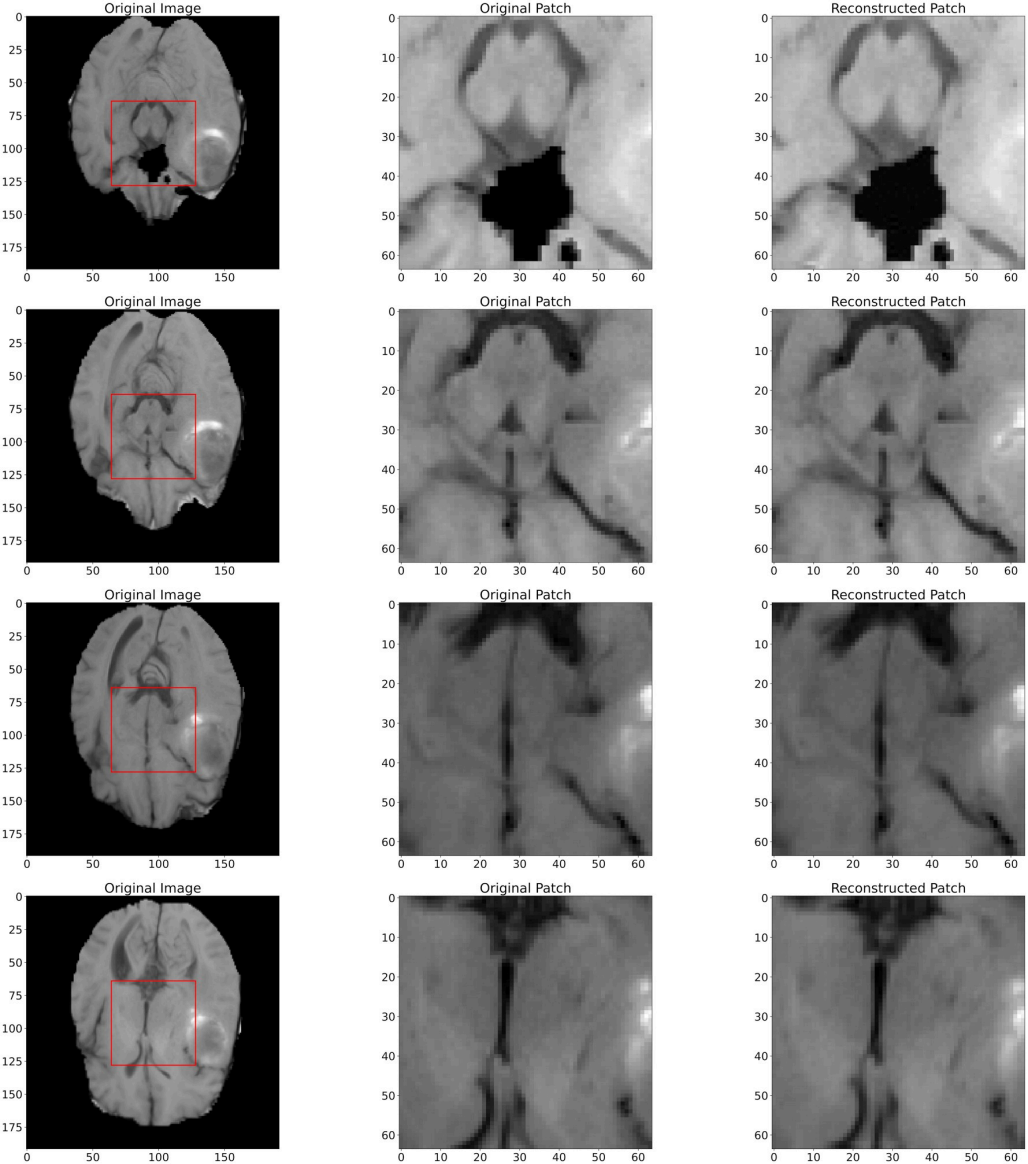
The Left column shows the different original slices of the volume with sizes of (155, 240, 240); the middle column shows the labelled patches of the slices with sizes of (64, 64, 64); the right column shows the reconstructed patches by our architecture.

Moreover, it is further illustrated by Eq 9. We show the three sets of marginal distributions concerning dimensions (NC, DS, SN), in Figs 3–5, respectively. If CR is decreased, the SIREN size (SN) or channel number (NC) can be increased. However, the reconstruction quality (i.e. PSNR or SSIM) shows a slight improvement. Thus, enlarging the model size or channel number will not significantly improve reconstruction quality. Additionally, compared to other existing approaches in Table 8, our architecture excels in maintaining a low Bitrate(bpp), ensuring that the compressed file size is significantly smaller. Our results (PSNR and SSIM) are still comparable with those of the “3D-VOI-OMLSVD [34]”. Fig 6 further shows the reconstructed slices of volume data.

**Fig 7:**
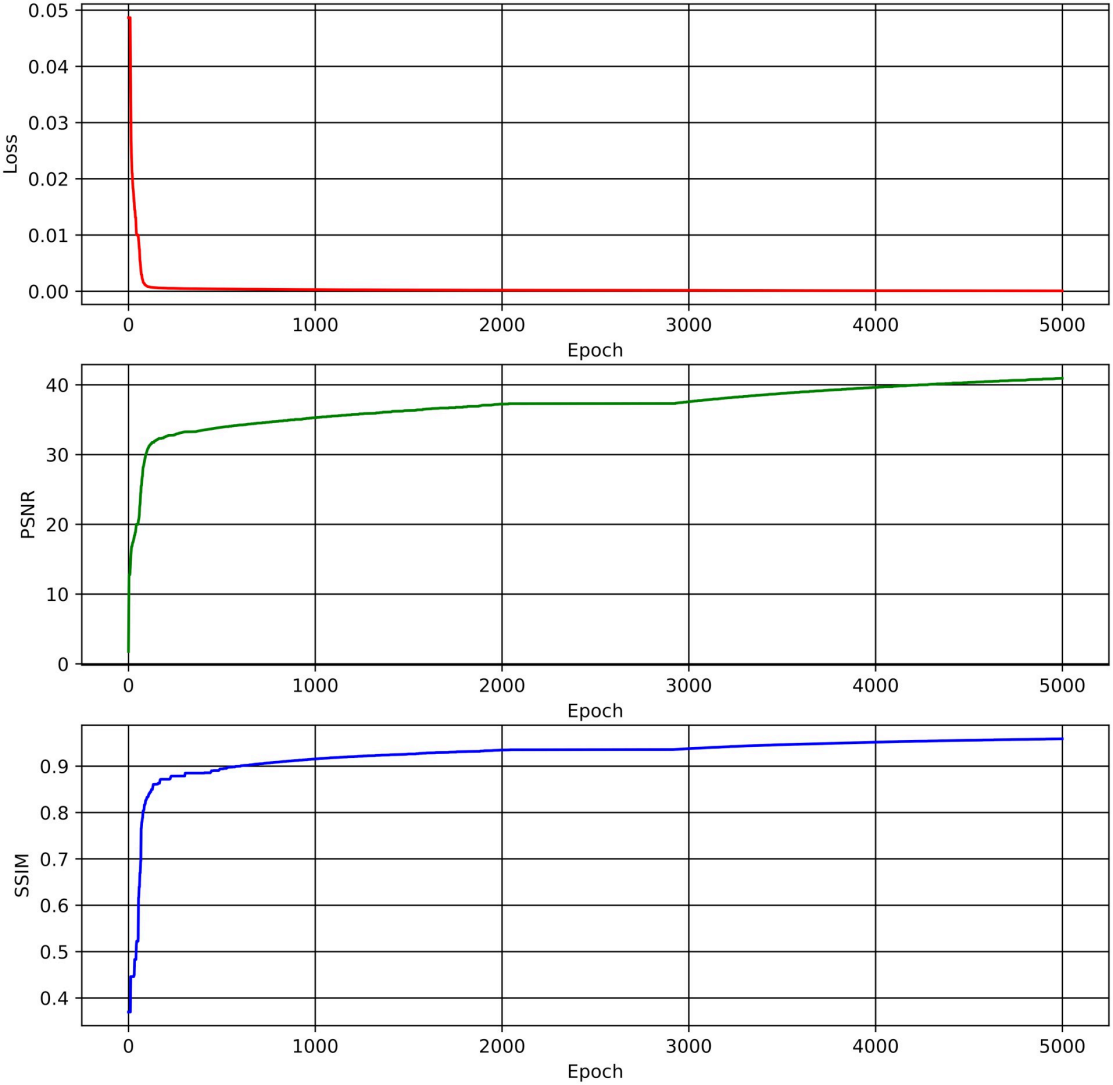
Training procedure of the architecture according to the trade-off point setting.

Additionally, Fig 7 shows a steady optimisation process over 5000 epochs, with continuous improvements in reconstruction accuracy and structural similarity. The PSNR curve exceeds 40 dB, indicating high reconstruction quality with minimal error. The SSIM curve approaches 0.96, demonstrating the model’s effectiveness in preserving perceptual and structural fidelity. The steady decrease in the loss function, alongside the PSNR and SSIM improvements, confirms effective convergence. These results, consistent with the final performance metrics in Table 8, highlight the architecture’s ability to balance compression efficiency and high-quality reconstruction, making it ideal for medical imaging.

**Table 8:** Comparison of our techniques with other state-of-the-art methods in terms of PSNR and SSIM in volume reconstruction.

Method	avg PSNR ↑	avg SSIM ↑	CR(%)↑	Bitrate(bpp) ↓	GPU(GB) ↓
Single SIREN [9]	40.008	0.947	67.062	10.348	3.390
Devadoss et al. [33]	34.1098	-	78.16	4.580	-
MVAR [32]	40.050	-	90.00	-	-
3D-VOI-OMLSVD [34]	**42.04**	**0.978**	89.17	2.54	-
aiWave-heavy [36]	39.00	-	-	2.5	-
Block CS [35]	30.86	0.7489	50.00	-	-
EZW with Haar [37]	30.15	-	40.31	-	-
Our Architecture	40.052	0.961	**97.578**	**0.775**	**0.769**

**Remark:** The proposed trade-off point approach serves as a pragmatic optimisation strategy. In the context of the compression problem, it is essential to balance various requirements, including downsampling scales, INR module size, SR module structure, etc., rather than overemphasising one or two factors. The trade-off point approach addresses this challenge by elegantly optimising the parameters involved.

## 5 Conclusion and future work

In this paper, we proposed an innovative architecture that integrates available deep-learning techniques with a focus on compressing volume data while maintaining high reconstruction fidelity. One notable aspect of our approach is the utilisation of emerging deep learning technologies, which have witnessed rapid development in recent years. We emphasised the importance of carefully considering various factors such as network architecture, computational efficiency, and reconstruction accuracy when designing and implementing the end-to-end solution. To this end, we proposed the end-to-end network architecture for volume data compression and developed the trade-off approach to determine optimal settings for individual modules, which is a practical method to balance performance considerations in the context of medical visualisation tasks.

### 5.1 Limitations

#### 5.1.1 Generalisation to diverse medical datasets

Applying the proposed end-to-end architecture to various volume datasets requires significant retraining time for each dataset individually, as there is no fine-tuning strategy in place to speed up this process.

#### 5.1.2 Time complexity of trade-off point approach

The trade-off point method necessitates sampling the model’s performance across different architecture settings, which is highly time-consuming.

### 5.2 Future work

Beyond the realm of compression, visualising over-large medical volume data through real-time rendering is meaningful. Compression with rendering could enable real-time visualisation of such over-large volume data. In future work, we intend to focus on volume-rendering techniques that leverage implicit neural representations. This research direction shows significant promise for advancements in the field of visualisation.
