# Supplementary material for: An end-to-end implicit neural representation architecture for medical volume data
Source: PLoS One. 2025 Jan 3;20(1):e0314944. doi: 10.1371/journal.pone.0314944 (PMC11698368; doi:10.1371/journal.pone.0314944)
Supplement: Attachment — Submitted filename: response to reviewers.pdf [file pone.0314944.s001.pdf]

We would like to express our sincere gratitude to the reviewers for their insightful comments and constructive feedback. Your valuable input has significantly contributed to the improvement of our work. Thank you for your time and effort in reviewing our manuscript. The following sections will address your concerns in detail.

Reviewer #1 Comments and Responses

1. **comment:**A preliminary theoretical analysis of the proposed methodology motivates its rationale. Also, this manuscript has many problems, such as structure, contribution, proposed method, etc. So, In my opinion, this is not suitable for publication.

   Response: Thank you for your valuable feedback on our manuscript. We sincerely apologise for the initial structural and wording shortcomings that may have impacted the clarity and quality of our work. In response to your comments, we have made substantial revisions to address the issues you raised. We have carefully reworded the Abstract, Introduction, and Methodology sections to enhance their clarity and readability. Additionally, we have revisited our experimental setup and findings to ensure that our results are presented in a more convincing manner. To further strengthen our manuscript, we have incorporated 6 recent references and conducted a comprehensive comparison with state-of-the-art methods. The updated results, particularly those presented in Table 3, now clearly demonstrate the advantages of our proposed method over existing alternatives. In essence, we have undertaken a comprehensive revision that amounts to a near rewrite of our paper. We are confident that these changes significantly improve the quality and clarity of our work, making it more suitable for publication. We appreciate the time and effort you have invested in reviewing our manuscript and providing constructive criticism. We hope that our revisions will meet your expectations and satisfy the criteria for publication in PLOS ONE. Thank you once again for your consideration.

Reviewer #2 Comments and Responses

1. **Comment:** The professional English editing is recommended. The authors should get editing help from someone with full professional proficiency in English.
   Response: The revised manuscript has undergone paraphrasing and enhancement for language clarity.

2. **Comment:** What is the main difference or importance of the proposed methods and the other state-of-the-art?
   Response: Our approach involves applying the proposed trade-off point

method to optimise the settings of deep networks. This optimisation strategy allows for a balanced consideration of multiple requirements, rather than overemphasizing one or two factors. For instance, the size of the INR module, the structure of the SR module, and the downsampling scales are all taken into account. The resulting end-to-end architecture delivers impressive outcomes. In our revision, we have included more comparisons with state-of-the-art methods in Table 3. The results indicate that our method surpasses others in performance.

3. **Comment:** The abstract should reflect the contributions of the manuscript. I suggest rewriting it.
   Response: Thanks for your suggestion. The abstract has been rewritten accordingly in our revision.

4. **Comment:** More recent references in the context of this study need to be added and discussed in the manuscript. For example, "A Secure Visual Framework for Multi-index Privacy Protection Evaluation in Networks, doi 10.1016/j.dcan. 2022.05.007."
   Response: Thank you for your suggestion. Upon review, the paper you mentioned appears to be unrelated to medical image compression, and as such, it has not been included in the references of our revised manuscript. However, we have added six recent papers to our references and conducted a more comprehensive comparison, as presented in Table 3 of our revision.

5. **Comment:** The Conclusion section should point out the potential disadvantages and possible future research directions of the manuscript. How this work can be extended in future?
   Response: Thanks for your suggestion. We have rephrased the conclusion in our revision. Our future work aims to integrate compression and rendering together into a end-to-end deep network.
