# Supplementary material for: An end-to-end implicit neural representation architecture for medical volume data
Source: PLoS One. 2025 Jan 3;20(1):e0314944. doi: 10.1371/journal.pone.0314944 (PMC11698368; doi:10.1371/journal.pone.0314944)
Supplement: Attachment — Submitted filename: Response to Reviewers.docx [file pone.0314944.s002.docx]

Dear peer-reviewers,

Many Thanks for your encouraging comments!

We read your comments carefully and update our submission accordingly. We write a response letter to address our modification as below.

If further query, let me know please.

Best regards,

Hongchuan Yu

-----------------------------------------------------------

Reviewer #1

1. The authors should clearly state the limitations of the proposed method in other real applications.

- Response:

*- The proposed method’s limitations have been stated in the conclusion in the revision [349-356]*

2. The abstract section is fragile. Please rewrite it, explain the result obtained and contribution, improve a proposed method, and delete unnecessary information.

- Response:

*- The abstract has been rewritten in the revision*

3. Proofread the manuscript carefully to eliminate any grammatical errors or typos and ensure clarity and coherence in writing. Additionally, adhere to the formatting and style guidelines specified by the target journal or publication venue to enhance the professionalism of the manuscript.

- Response:

*- The grammatical errors or typos have been corrected in the revision*

*- Guidelines were checked*

4. I suggest the authors add a table at the end of the literature review and compare the reviewed papers to clarify the research gap better.

- Response:

*- The suggested table has been added as Table 1 in the revision*

5. Please write your contribution to this paper in the Introduction section.

- Response

*- The contributions mentioned before, but they have been expanded for clarification in the revision.[31-37]*

6. Expand the critical results in the conclusion. Focus on the main developments in the finale. Also, write the main contributions in the conclusion.

- Response:

*- The conclusion has been rewritten accordingly in the revision [338-348]*
7. Numerical results are good enough, but more explanations are required to analyse each figure presented.

*- Response*

*- In Find a trade-off architecture by trade-off point approach subsection, we added Fig 7 to show the training procedure of the end to end architecture. Moreover, we also rewrote the Materials and Experimental results Section to explain each figure and Table in details in the revision. [313-330]*
8. The simulation section needs to be more detailed. The authors should provide more informatio about the data they employed and the simulation process.

- Response:

*- The required explanations about simulation process have been added in the Materials and Experimental Results section. Data and implementation setup subsection has been rewritten in the revision. [234-260]*

9. Please Change the “conclusion” section to “ Conclusion and Future Work” and write future work.

- Response

*- It has been changed in the revision [337]*
10. All figures are of low quality, so please improve all of them.

- Response

*- The PACE tool standardised the images according to the guidelines. But this time for assurance, the original figures' configurations and quality were adapted to the guidelines with a maximum resolution of 600 dpi.*

Reviewer #2: 
1. The professional English editing is recommended. The authors should get editing help from someone with full professional proficiency in English.

- Response:

*- The revision has been proofread to correct errors.*
2. The abstract contains a large amount of information. It is recommended to simplify the language and highlight the innovative points and main results of the study. It is suggested to update some higher quality or newer literature. For example, the introduction of convolutional neural networks can be referenced as "A Novel Centralized Federated Deep Fuzzy Neural Network with Multi-objectives. Neural Architecture Search for Epistatic Detection, DOI: 10.1109 / TFUZZ. 2024.3369944 ', Fourier Transform technique introduction can be quoted "An IoMT enabled deep learning framework for automatic detection of fetal QRS: A solution to remote prenatal care, DOI10.1016 / j.j ksuci. 2022.07.002.

- Response

- *These missed reference have been cited in the revision. [11-12]*
3. The figures included in the manuscript are not sufficiently clear. High-resolution images should be provided to ensure that all details are visible and can be thoroughly examined by readers.

- Response

*- The PACE tool standardised the images according to the guidelines. For assurance, the original high quality figures were uploaded instead in the revision.*

4. The introduction should provide a clearer overview of the motivation, objectives, and main contributions of the study. Additionally, the limitations of existing technologies and how your work addresses these gaps should be briefly discussed.

- Response:

*- The introduction has been rewritten accordingly and for clarification, table 1 has been added to show the gaps in the revision.*
5. Please outline your main contributions more clearly in the introduction and conclusion sections. This should include the specific innovations of your proposed end-to-end architecture and the advantages over traditional methods.

- Response

*- The contributions have been explicitly mentioned in introduction and conclusion in the revision.[31-37][338-348]*

6. Your proposed end-to-end architecture requires a more detailed description.

- Response

- *The Proposed End-to-End architecture subsection has been updated accordingly and Data and implementation setup subsection has been updated as well in the revision. [100-104]*

7. The results section requires more details to substantiate your claims. For instance, for the performance evaluation of each module, more quantitative data and visualizations should be provided.

- Response

*- In Find a trade-off architecture by trade-off point approach subsection, we added Fig 7 to show the training procedure of the end to end architecture. Moreover, we also rewrote the Materials and Experimental results Section to explain each figure and Table in details in the revision.[265-268][272-274][287-293]*

8. The selection of the dataset, experimental setup, and evaluation metrics need more detailed descriptions to ensure the reproducibility and validity of the results.

- Response

*- More details have been added to the Data and implementation setup subsection in the revision. [231-234]*
9. Please ensure that all relevant and up-to-date literature is cited and that the citation format adheres to the journal's requirements.

- Response

*- The citations and their format have been reviewed in the revision.*
10. Discuss in more detail within the text how your work relates to existing technologies. This includes a comparison of your work with the current state-of-the-art and where your work offers improvements.

- Response

*- Table 7 shows the comparison with SOTA and has been updated in both caption and description in text in the revision.[319-321]*
